# Intramedullary spinal cord metastasis from prostate carcinoma: a case report

**DOI:** 10.1186/1752-1947-6-139

**Published:** 2012-06-01

**Authors:** Robert E Lieberson, Anand Veeravagu, Jan M Eckermann, James R Doty, Bowen Jiang, Russell Andrews, Steven D Chang

**Affiliations:** 1 Department of Neurosurgery, Stanford University Medical Center, Stanford, CA, USA; 2 NASA-Ames Research Center, Mountain View, CA, USA; 3 Department of Neurosurgery, Robert C and Jeannette Powell Professor in the Neurosciences, Stanford University, 300 Pasteur Drive, Room R-225, Stanford, CA, 94305, USA

## Abstract

**Introduction:**

Although vertebral and epidural metastases are common, intradural metastases and intramedullary spinal cord metastases are rare. The indications for the treatment of intramedullary spinal cord metastases remain controversial. We present the first biopsy-proven case of an intramedullary spinal cord metastasis from adenocarcinoma of the prostate.

**Case presentation:**

Our patient was a 68-year-old right-handed Caucasian man with a Gleason grade 4 + 3 prostate adenocarcinoma who had previously undergone a prostatectomy, androgen blockade and transurethral debulking. He presented with new-onset saddle anesthesia and fecal incontinence. Magnetic resonance imaging demonstrated a spindle-shaped intramedullary lesion of the conus medullaris. Our patient underwent decompression and an excisional biopsy; the lesion’s pathology was consistent with metastatic adenocarcinoma of the prostate. Postoperatively, our patient received CyberKnife^®^ radiosurgery to the resection cavity at a marginal dose of 27Gy to the 85% isodose line. At three months follow-up, our patient remains neurologically stable with no new deficits or lesions.

**Conclusions:**

We review the literature and discuss the indications for surgery and radiosurgery for intramedullary spinal cord metastases. We also report the novel use of stereotactic radiosurgery to sterilize the resection cavity following an excisional biopsy of the metastasis.

## Introduction

Adenocarcinoma of the prostate affects up to 70% of men over 80 and is the second leading cause of cancer-related death in men [1]. Skull and spinal metastases occur in up to 10% of patients with prostate cancer but intradural lesions are uncommon. We are aware of no prior reports of biopsy-proven intramedullary spinal cord metastases (ISCMs) due to prostate cancer. Conventional radiation has been the historic treatment of choice for radiosensitive ISCMs, with surgery preferred in select cases [2,3]. Stereotactic radiosurgery (SRS) has been discussed as a primary treatment for ISCMs [4,5]. Furthermore, SRS is a favored alternative to postoperative whole brain radiotherapy (WBRT) following the resection of a brain metastasis [6]. We report the first biopsy-proven case of an ISCM from prostate cancer and discuss a novel treatment regimen: the use of a radiosurgical boost to the resection cavity of an ISCM as an alternative to postoperative spinal radiation for a relatively radioresistant metastasis.

## Case presentation

A 68-year-old, right-handed Caucasian man was diagnosed with Gleason grade 4 + 3 adenocarcinoma of the prostate in March 2009. A partial prostatectomy and pelvic node dissection was completed, and our patient started androgen blockade with leuprolide (Lupron), dutasteride (Avodart) and bicalutamide (Casodex). His prostate specific antigen (PSA) levels decreased from an initial value of 41.6 to 0.07 ng/ml in April 2010. In May 2010, one month prior to admission, a transurethral debulking of his prostate was completed for difficulty in initiating a urine stream and an indwelling Foley catheter was left in place. In the 48 hours prior to admission, he developed a worsening saddle anesthesia. On the day of admission, June 10 2010, he described increasing headache as well as new-onset fecal incontinence but denied weakness. His past history, family history and a review of his systems were not significant.

On admission, his mental status, cranial nerve and upper extremity examinations were unremarkable. A motor examination was intact in his legs but there was significant numbness below his ninth thoracic vertebra. Reflexes were absent in his lower extremities. Rectal sensation and tone were absent.

**Figure 1:**
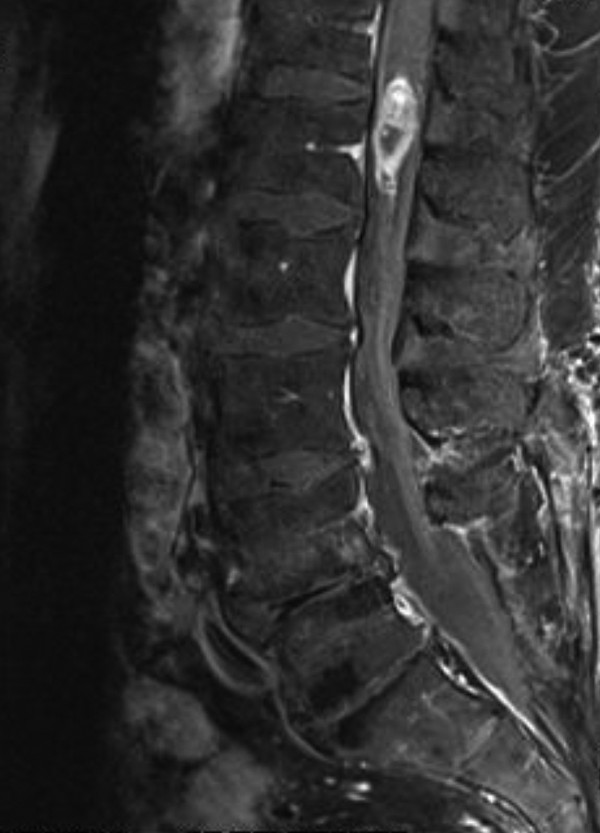
Sagittal contrast-enhanced T1-weighted magnetic resonance image.

**Figure 2:**
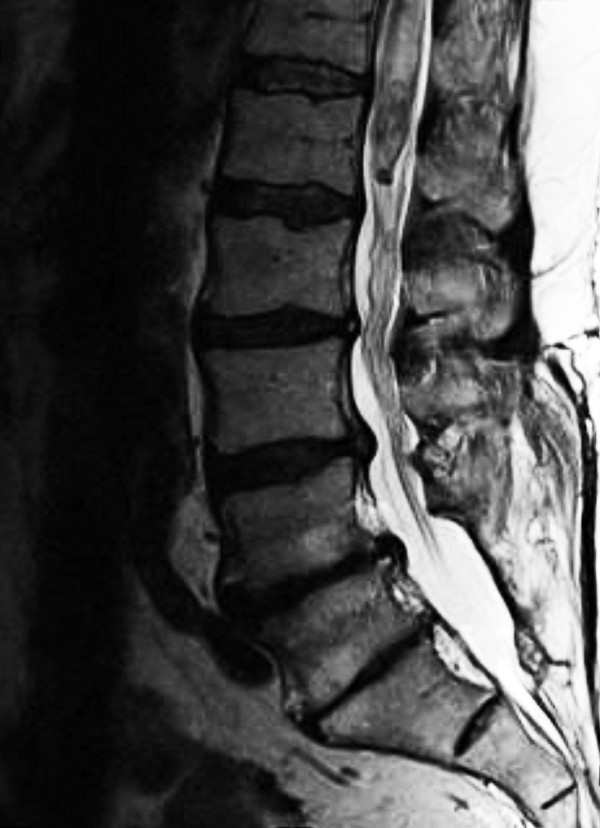
Sagittal T2-weighted magnetic resonance image.

**Figure 3:**
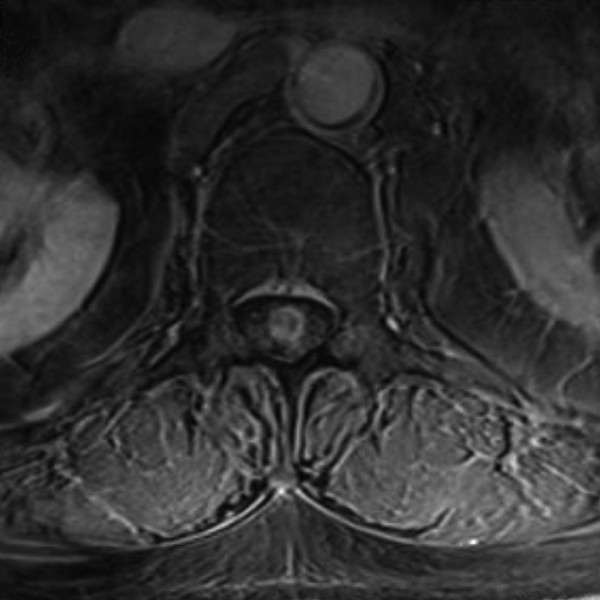
Axial contrast-enhanced T1-weighted magnetic resonance image.

Magnetic resonance imaging scans of his brain and thoracic and lumbar spine showed two irregularly enhancing lesions. The first, 2cm in diameter, was in the region of the fourth ventricle but was without secondary hydrocephalus. The second was a spindle-shaped intramedullary lesion of the conus medullaris (Figures 1, 2 and 3). The lesion was approximately 2cm in the vertical extent and appeared partially cystic.

Our differential diagnosis included ependymoma, astrocytoma, a metastasis from the known prostate cancer or a metastasis from an undiscovered second primary tumor.

Because of our patient’s increasing neurological deficit, and given the uncertainty of the diagnosis, a decompression and an excisional biopsy were undertaken on the day of admission. With somatosensory and motor evoked potential monitoring, a twelfth thoracic to first lumbar vertebral laminectomy was completed. The level of the lesion was confirmed using intraoperative ultrasound. On opening the dura, abnormal appearing tissue was immediately identified involving the posterior surface of the conus. A small biopsy was consistent with malignant tissue. A more aggressive resection to the edges of the abnormal appearing tissue was completed and his cord appeared adequately decompressed. The closure was uneventful.

**Figure 4:**
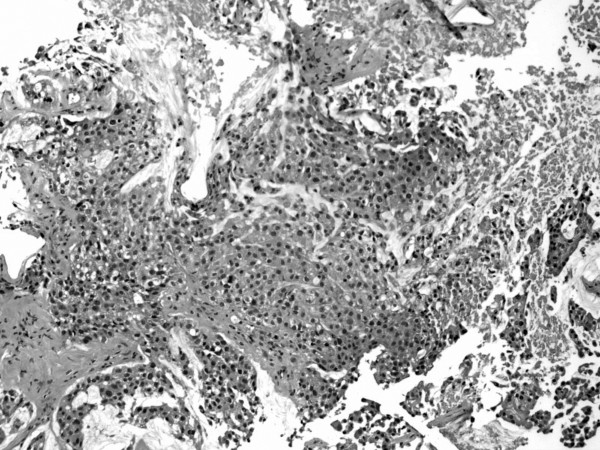
Hematoxylin and eosin stained section.

On microscopy, the lesion showed sheets and nests of cells with abundant cytoplasm and prominent nucleoli. Mitoses were common. Intracytoplasmic and extracellular mucin was demonstrated. AEI/AE3 cytokeratin, CAM5.2, CK7, and CK20 were positive. PSA, Prostatic specific acid phosphatase (PSAP), thyroid transcription factor-1 (TTF-1), caudal type homeobox-1 (CDX-1), and S100 were negative. The pathology was consistent with metastatic adenocarcinoma of the prostate (Figure 4).

Following surgery, his sensory changes improved and the motor examination remained intact. His bowel and bladder function remained poor. The intracranial lesion and the resection cavity in the conus were both treated with CyberKnife^®^ radiosurgery. The spinal resection cavity, contoured to the edge of the enhancing tumor on a postoperative scan, was treated to a marginal dose of 27Gy at the 85% isodose line in three sessions. Our patient remains neurologically stable three months after treatment, with no new neurological deficits and no new lesions.

## Discussion

Although vertebral and other epidural metastases are common, intradural metastases are rare. ISCMs represent only 0.9% to 2.1% of autopsy cases in patients with cancer and only 5% of these are identified antemortum [2]. Adenocarcinoma of the prostate affects up to 70% of men over 80 and is the second leading cause of cancer-related death in men [1]. Meningeal carcinomatosis, brain metastases and intradural extramedullary spinal lesions are uncommon. One prior case report discussed a possible prostate ISCM [7]. To date, however, there have been no reports of biopsy-proven prostate cancer metastatic to the spinal cord.

The diagnosis of ISCMs is usually radiographic. Myelography may show a fusiform enlargement of the cord but is non-diagnostic in 40% of cases. Although cerebral spinal fluid (CSF) is frequently abnormal, malignant cells are present in only the minority of cases. Interestingly, Schaller *et al*. demonstrated in a case report that PSA in the CSF may be a useful diagnostic tool for detecting intradural prostate metastasis [8]. There are currently no published treatment guidelines for this ISCM, but options include conventional radiation, SRS, surgery, steroids and chemotherapy.

Conventional radiation has been the historic treatment of choice [2,3,9]. It is effective for radiosensitive ISCMs but not radioresistant tumors, such as prostate tumors. Surgery is considered for the palliation of pain or the prevention of paraplegia when the medical condition of the patient is appropriate [3,10]. Steroids are appropriate as an adjunct. Chemotherapy has been used in combination with surgery or radiation for sensitive tumors, such as small-cell carcinomas, while SRS is increasingly a consideration for ISCMs.

Several authors have described SRS as a primary treatment for ISCMs. Parikh and Heron [4] described a single patient with a renal cell metastasis treated with CyberKnife^®^, at a 15Gy marginal dose in three fractions. Their patient had no recurrence in the 26 months of follow-up. Shin *et al*. [5] presented nine patients, of whom six had ISCMs. As in the patient of Parikh and Heron, all of Shin’s patients also had brain metastases. Various linear accelerator systems were used. A median marginal dose of 14Gy was delivered in one fraction. The neurological examinations of five of the six patients improved and one remained unchanged. In five of six patients with an ISCM, local control was achieved. Of note, in both studies the cord dose exceeded the 10Gy maximum dose that is considered safe. In neither case reports were radiation-related complications reported.

An SRS boost to the resection cavity is an appropriate postoperative alternative to WBRT in patients with brain metastases. Kim *et al*. [11] described the use of re-resection coupled with Gamma Knife SRS in 79 patients whose brain metastases recurred after both resection and postoperative WBRT. Their local control rate, in this group who had failed surgery and WBRT, was 94.9% and the mean survival time was 17 months. Complications included radionecrosis in 5% to 10% of cases. Soltys *et al*. [12] presented 72 patients treated with CyberKnife^®^ SRS after resection instead of WBRT. Local control was 86% in those treated to the margin of the enhancing tumor, but was 100% in those where the treatment volume extended 2mm beyond the edge of the contrast-enhancing resection cavity. A subsequent course of WBRT was required for other lesions in 19% of patients (in a mean of six months after SRS). Mean overall survival was 15 months but excluding non-neurological causes of death was 16.9 months. Hwang *et al*. [6] directly compared postoperative Gamma Knife SRS and WBRT in 43 patients (25 SRS and 18 WBRT). Local control was 100% for SRS-treated patients and 83% in the WBRT group. Median survival was 15 months for SRS-treated patients and 6.8 months for the WBRT group, but was not statistically different once distant recurrences were added to the model. SRS to the resection bed of brain metastases was therefore similar or superior in efficacy to postoperative WBRT, the neurological risks associated with WBRT were avoided and the long time required for treatment was decreased from weeks to five or fewer days.

At present, there is no study in the literature that directly compares success rates for radiotherapy alone, surgery alone or surgery plus radiotherapy for ISCMs. We feel it is likely that the efficacy of the various treatments of ISCMs would parallel that of brain metastases, and that a local boost to a resection cavity in the spinal cord should be as effective as conventional postoperative spinal radiation while potentially decreasing risk and minimizing the length of treatment. It also seems reasonable to use SRS for lesions, such as prostate lesions, that respond poorly to conventional radiation and are shown to respond well to SRS in the brain [10].

This patient had good residual lower extremity function and a lesion whose pathology was uncertain. Surgery was recommended to preserve function, was completed without incident and was followed by clinical improvement. After surgery, conventional radiation and SRS treatment were considered. An SRS boost to the resection cavity was recommended once the pathology confirmed the metastasis as from the prostate.

## Conclusions

Metastases to the intramedullary spinal cord are rare and typically occur late in the course of the disease. Surgery is appropriate for those patients who can tolerate the operation and who are in danger of permanent loss of function or who have intractable pain. After surgery, conventional spinal radiation has been provided to decrease the risk of local recurrence. There is risk to spinal radiation and not all tumors are radiosensitive. We propose that a local cavitary boost with SRS may be as effective as conventional radiation for some tumors while offering greater convenience and perhaps less risk.

## Consent

Written informed consent was obtained from the patient for publication of this case report and any accompanying images. A copy of the written consent is available for review by the Editor-in-Chief of this journal.

## Abbreviations

CSF, Cerebral spinal fluid; ISCM, Intramedullary spinal cord metastasis; PSA, Prostate specific antigen; SRS, Stereotactic radiosurgery; WBRT, Whole brain radiotherapy.

## Competing interests

The authors declare that they do not have any competing interests.

## Authors’ contributions

RL, AV, JE and JD collected, analyzed and interpreted the patient data, conducted the literature review and were the major contributors in manuscript drafting. BJ and RA provided critical revisions of the manuscript. RL, AV, JE, JD and SC participated in patient evaluation, administration of treatment and enrolling the patient. SC conceived of the study and participated in its design and coordination and helped to draft and review the manuscript. All authors read and approved the final manuscript.
